# Supplementary material for: COZOID: contact zone identifier for visual analysis of protein-protein interactions
Source: BMC Bioinformatics. 2018 Apr 6;19:125. doi: 10.1186/s12859-018-2113-6 (PMC5889581; doi:10.1186/s12859-018-2113-6)
Supplement: Supplementary file 2 — Software build. Executable binary file of the software tool. (ZIP 118,784 kb) [file 12859_2018_2113_MOESM2_ESM.zip › cozoid/platform/docs/testng-6.5.1-javadoc.zip!/org/testng/IMethodInstance.html]

IMethodInstance (TestNG)


---


|  |  |  |
| --- | --- | --- |
| **Overview** \| **Package** \| **Class** \| **Tree** \| **Deprecated** \| **Index** \| **Help** | |  |
| **PREV CLASS**   **NEXT CLASS** | **FRAMES**    **NO FRAMES**     **All Classes** |  |
| SUMMARY: NESTED \| FIELD \| CONSTR \| METHOD | DETAIL: FIELD \| CONSTR \| METHOD |  |


---


## org.testng Interface IMethodInstance

---

``` public interface IMethodInstance ```

This interface captures a test method along with all the instances it should
be run on.

---

| **Method Summary** | |
| --- | --- |
| `java.lang.Object` | `getInstance()` |
| `java.lang.Object[]` | `getInstances()`             **Deprecated.** *Use getInstance()* |
| `ITestNGMethod` | `getMethod()` |

**Method Detail**

### getMethod

```
ITestNGMethod getMethod()
```

---


### getInstances

```
java.lang.Object[] getInstances()
```

:   **Deprecated.** *Use getInstance()*

---


### getInstance

```
java.lang.Object getInstance()
```


---


|  |  |  |
| --- | --- | --- |
| **Overview** \| **Package** \| **Class** \| **Tree** \| **Deprecated** \| **Index** \| **Help** | |  |
| **PREV CLASS**   **NEXT CLASS** | **FRAMES**    **NO FRAMES**     **All Classes** |  |
| SUMMARY: NESTED \| FIELD \| CONSTR \| METHOD | DETAIL: FIELD \| CONSTR \| METHOD |  |


---
